# A novel chitosan–collagen bilayer scaffold prevents contraction and accelerates cutaneous repair in a rat splint-skin model

**DOI:** 10.3389/fbioe.2026.1802308

**Published:** 2026-04-01

**Authors:** Priya Das, Matthew McGrath, Noof Sulaiman, Martin Maresch, Nigamananda Dey, Melvin Varghese Jacob, Mohammed Al Muharraqi, Shane Browne, Fergal J. O’Brien, Michael B. Keogh

**Affiliations:** 1 TERG Bahrain, School of Postgraduate Studies and Research, Royal College of Surgeons in Ireland, Manama, Bahrain; 2 Department of Anatomy and Regenerative Medicine, Tissue Engineering Research Group, Royal College of Surgeons in Ireland, Dublin, Ireland; 3 RMS Royal Medical Services, Riffa, Bahrain; 4 Charis Veterinary Clinic, Budaiya, Bahrain; 5 CÚRAM Research Ireland Centre for Medical Devices, University of Galway, Galway, Ireland; 6 Advanced Materials and Bioengineering Research Centre (AMBER), RCSI, Dublin, Ireland

**Keywords:** biomaterial, gene activated scaffolds, nanoparticle, pro-angiogenic, splint skin wound, UN SDG3

## Abstract

**Introduction:**

The treatment of chronic wound is extremely challenging and is often exacerbated by inflammation, poor angiogenesis and recurrent bacterial infections. To address this, we have developed a novel biomimetic bilayer three-dimensional scaffold with a chitosan-collagen upper epidermal layer, on top of a porous collagen-glycosaminoglycan dermal layer.

**Methods:**

In this study we assess this scaffold’s efficacy in a preclinical wound model. In addition, we examined the scaffold with the addition of plasmid DNA encoding pro-angiogenic stromal derived factor-1α (SDF-1α) and anti-fibrotic β-klotho in a splinted full-thickness skin wound model on young Sprague Dawley rats for 14 days.

**Results:**

All the scaffold groups showed uniform deposition of extracellular matrix and showed no signs of wound contraction unlike our ‘empty’ defect group. Both the ‘bilayer chitosan- collagen’ group and ‘gene activated group’ showed that the upper chitosan layer was filled with exudate, which dried over time and formed a protective scab that delaminated easily at day 14. Our Chitosan- collagen scaffolds showed a decrease in pro-inflammatory IL-1β, an increase in the pro-angiogenic CD31 and a decrease in pro-fibrotic α-SMA protein expression. We showed enhanced pro-angiogenic and reduced pro-fibrotic expression with the addition of SDF and Klotho plasmids respectively (p < 0.01); however, the rate of wound healing was reduced with gene activation.

**Discussion:**

While the chitosan layer of the bilayer scaffold does not integrate into the wound bed it does form a protective covering with enhanced anti-inflammatory cues that support the lower integrating dermal collagen layer yielding optimal anti-fibrotic wound healing. These properties highlight the potential of this chitosan-collagen bi-layered scaffold, suggesting its suitability for promoting enhanced healing of chronic wounds in clinical settings.

## Graphical Abstract

Created in BioRender. Bahrain team 1, R. (2025) https://BioRender.com/4pugypi.

**Figure:**
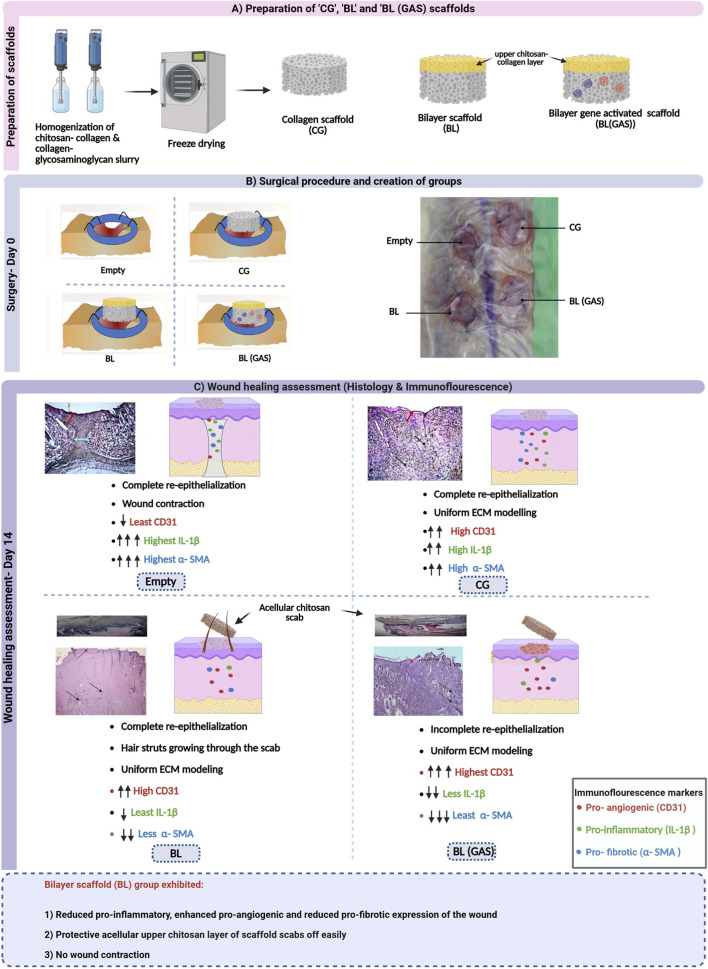

## 1 Introduction

Chronic wounds develop due to underlying clinical conditions such as diabetes, vascular diseases, aging and genetic disorders (hemoglobinopathies), continue to be a major cause of morbidity and medical burden associated with its long-term care. Among these, diabetic foot ulcers (DFUs) are particularly concerning, as they increase mortality risk by more than 2.5-fold in diabetic patients and frequently leads to infection in nearly half of the cases, with about one-fifth progressing to amputation. The timely organised sequel of normal cutaneous wound healing involving haemostasis, inflammation, proliferation, and remodelling goes awry in chronic wounds especially due to stalled inflammation phase, lack of angiogenesis and the unhealed wound continuously being prone to more infections (Armst et al., 2017; Armst et al., 2023; Rodrigues et al., 2019; Zhang et al., 2020). Recurrent bacterial infections and continuous activation of toll-like receptors sustain a pro-inflammatory environment and hence disrupts wound healing and promotes chronic wound development (Versey et al., 2021). Long term use of antibiotics which is a part of clinical management further poses the risk of developing drug resistance in the comorbid subjects (Punjataewakupt et al., 2019).

Management of chronic wounds such as diabetic wounds warrants a strategic development of a sustainable biomaterial (Wong and Gurtner, 2012). Over the decades tissue engineering approaches have enabled the evolution of 2D wound dressing to 3D biomaterials/scaffolds/hydrogels loaded with or without cells and biomolecules (proteins/DNA/RNA for growth factors, cytokines) to promote tissue regeneration and programmed wound healing. Recent reviews consistently highlight that biomaterial-based scaffolds have the ability to enhance angiogenesis, modulate inflammation, and accelerate tissue regeneration; however, their lack of inherent antimicrobial capacity remains a major limitation in addressing chronic wound infections (Chakrapani et al., 2025; Aiman et al., 2022; Shen et al., 2024). Antimicrobial chemicals such as gentamycin sulphate, polylysine, silver nanoparticles, iodine, polyhexanide, etc., have been incorporated in biomaterials and are widely tested as wound dressings (Shen et al., 2024; Liang et al., 2024; Homaeigohar and Boccaccini, 2020). Recently research has also examined the use of natural agents such as manuka honey, plant extracts or even crustacean polysaccharides like chitosan for their antimicrobial wound healing potential (Bulman et al., 2015; A et al., 2025).

In our laboratory we have developed a bilayer chitosan-collagen scaffold skin graft substitute (McGrath et al., 2023). The unique design has an upper chitosan- collagen film layer that provides antimicrobial protection against infection, combined with a lower collagen–glycosaminoglycan (CG) porous layer that promotes wound healing by allowing cell attachment and migration. *In vitro* results showed that the upper chitosan-collagen layer of the bilayer scaffolds successfully restricted the entry of microbes to the lower collagen layer and the inner layer of the scaffold supported cell attachment, survival and angiogenic potential. Based on the *in vitro* findings, the *primary aim* of this study was to investigate the *in vivo* wound healing efficacy of the bilayer scaffolds to support skin regeneration (McGrath et al., 2023).

Functionalizing biomaterials prior to their application to the wound provides biological cues that promote dermal tissue alignment and enhance the quality of the neodermis (Wong and Gurtner, 2012). Supporting angiogenesis and controlling fibrosis are also vital for proper wound repair, especially in chronic cases (Singer, 2022). During the proliferative phase of wound healing, angiogenesis plays a crucial role. SDF-1α, a key chemokine, facilitates this process by attracting endothelial progenitor cells to the wound bed and stimulating angiogenic signaling via upregulating vascular endothelial growth factor and platelet endothelial cell adhesion molecule expression (Deshane et al., 2007). Regulating fibrosis is equally essential for guiding the wound healing process toward a more regenerative outcome. β-Klotho is an endogenous protein and is known for its anti-aging role and in regulating fibrosis by directly inhibiting TGF-β, WNT and FGF2 signalling (Zhao et al., 2024). Our previous *in vitro* studies have demonstrated that gene-activated collagen scaffolds incorporating SDF-1α and β-Klotho plasmids enhance angiogenesis, regulate fibrosis, and accelerate wound healing (Laiva et al., 2021; Laiva et al., 2024; Suku et al., 2020). Increased vascularization was also noted in the neodermis of the SDF-1α activated collagen scaffolds *in vivo* as reported in our previous study (Das et al., 2025). This rationale supports the *secondary aim* of this *in vivo* study to determine the therapeutic potential of dual gene-activated bilayer chitosan- collagen in promoting robust neo-vascularization while simultaneously minimizing fibrotic tissue formation, thereby offering a more effective approach for chronic wound repair.

## 2 Methods

### 2.1 Fabrication of bilayered scaffolds

A multistep process was involved in the fabrication of the bilayered scaffold. A collagen/chitosan film is subsequently added to the collagen chondroitin-6-sulfate (CG) slurry and is combined via lyophilization. Briefly, a solution of 0.75% w/v chitosan and 0.5% w/v type 1 collagen was made in 0.05 M acetic acid. The solution was blended and degassed prior to being air dried for 60 h. Similarly, collagen (CG) slurry was prepared in 0.0.5M acetic acid by mixing 0.5% w/v of type I bovine tendon collagen and 0.05% w/v of chondroitin-6-sulfate. The CG slurry was stored at 4 °C until use. Using lyophilization and optimized freeze-drying process, the chitosan films were combined with the CG slurry and were fabricated into three-dimensional scaffolds as previously described (McGrath et al., 2023; Murphy et al., 2010). Cylindrical scaffolds (8 mm diameter and 4 mm in height) were punched out from the sheets. The mechanical stability of the bilayered scaffold was further increased by using two-hour treatment with chemical cross-linkers 14 mM N-(3-Dimethylaminopropyl)-N′-ethylcarbodiimide hydrochloride and 5.5 mM N-Hydroxysuccinimide (EDC/NHS, Sigma, UK) solution and washed with phosphate buffered saline (PBS).

### 2.2 Preparation of gene-activated scaffold (GAS)

Cylindrical cross-linked scaffolds (8 mm diameter and 4 mm in height) were washed with PBS (Gibco, UK) As described in the previous studies, a polyplex particle with a N/P ratio of 10 was fabricated by combining 25.8 µL of 0.01% (w/v) of cationic 25 kDa polyethyleneimine (PEI) (Sigma-Aldrich, Dublin, Ireland) with 1 μg anionic pDNA SDF1α and 1 μg β-klotho plasmids SantaCruz Biotechnologies, USA and SinoBiological Beijing, China) in 20.2 µL of endotoxin free water (Laiva et al., 2024).

### 2.3 Animal handling

The study was approved by the RCSI Bahrain Ethics Committee REC/2020/41/4th-Oct2023 and Bahrain Defense Forces hospital ethics no. BDF/R&REC/2023-714. All the animals were handled as per international animal welfare standards. All the animals were inspected by a licenced independent veterinarian and were caged separately in the animal holding unit after a 10-day isolation period post-ivermectin treatment. The animals were maintained in a room with optimum temperature (22 °C ± 1 °C), humidity (50%–60%) and with 12/12 h of light/dark cycle. Microchips were used for accurate identification of each animal, which was implanted subcutaneously in the interscapular area using a minimally invasive technique (Ball et al., 1991).

### 2.4 Wound induction and surgery

**FIGURE 1:**
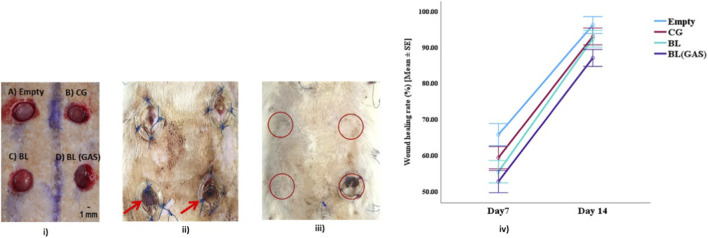
**(i)** Wound size at Day 0- Four full-thickness splint skin defects on the dorsal skin of young rats with, (A) upper left is “empty” group, (B) upper right is collagen “CG” group (C) lower left is chitosan-collagen bilayer ‘BL’ group, and (D) lower right is chitosan- collagen bilayer group activated with plasmids of pSDF1α and pβ-Klotho “BL (GAS)”; **(ii)** Wound size of rats at Day 7 post-surgery. Thick scab over the wounds at lower left and lower right sites corresponding to “BL” and “BL (GAS)” were noted (red arrows); **(iii)** Wound size of rats at Day14 post-surgery; **(iv)** Wound shrinkage rate (%) presented as Mean ± SE of all the groups from day 7 to day 14.

Seven male Sprague Dawley rats (10–12 weeks old) were used in this study and all the surgical procedures adhered to ethical standards. Anaesthesia was induced with 4%–5% isoflurane in a gas chamber and maintained at 2%–3% via a nose cone. The dorsal region was shaved, disinfected with 10% povidone-iodine, and animals were placed on pre-warmed heating pads in prone position. Continuous monitoring of vital parameters (respiratory rate, pulse, temperature) was performed to maintain the physiological stability. Maintaining 1.5 cm apart, four 8-mm full thickness wound biopsies were created using sterile punches on the dorsal surface of the rat’s skin. There were two biopsies each on left and right of the dorsal back reaching up to the hypodermal layer. After the excision of the skin flaps using an iris scissors, circular silicon splints (Grace biolabs, USA) with an inner diameter of 10 mm were placed on top of the wounds and sutured through the skin using 3-0 nylon sutures. The following biomaterials were loaded into the wounds: A) upper left was left empty and served as control (“empty” group); B) upper right with collagen scaffold (“CG” group) C) lower left with chitosan- collagen bilayer scaffold (“BL” group); and D) lower right chitosan- collagen chitosan gene activated (pSDF1α and pβ-Klotho) bilayer scaffold (“BL (GAS)” group) (Figure 1i).

Following surgery and loading the biomaterials, the wounds were covered with Opsite and dry gauze. To avoid dressing displacement and wound bed disruption an Elizabethan collar was placed around the head. Extra loops were created around the gauze for wound dressing change. Animals were then placed on recovery bed at 30 °C and were later caged separately to avoid cross- infections and monitored daily for 14 days.

### 2.5 Evaluation of wound healing

All the rats were monitored daily; routine monitoring of the animals included redressing and imaging the wound. Photographs were taken using a digital camera on day 7 and day 14. The wounds were monitored for their surface area and re-epithelization. Wound shrinkage rate was calculated as [(original area- epithelized wound area)/original area)] *100%.

### 2.6 Histological evaluation- H&E, Mason’s trichrome and Safranine O fast green staining

The sterile skin tissues were collected after the SD rats were terminated by cervical dislocation. Skin biopsies of the wound site and periphery were obtained using a sharp scalpel (2 cm square flaps). The collected skin was fixed overnight in 4% paraformaldehyde at 4 °C, processed using an automated tissue processor (Leica, Nusslock, Germany) and then embedded in paraffin. A rotary microtome was used to obtain 5 µm thick sections and were mounted on L-polysine coated glass slides (Sigma-Aldrich, France). Biopsies were deparaffinised followed by rehydration in alcohol prior to chemical staining with haematoxylin-eosin (HE) (Biognost, EU), or mason’s trichrome (Abcam, UK) for histological assessment. The wound scabs were stained with Safranine O Fast green staining to detect the presence of chitosan. Images were acquired by cellSens Imaging Software (Evident, Olympus Life Science Solutions, Japan).

Wound healing progression was evaluated using a on a qualitative scale criterion described by Arslantas et al. (2015), van de Vyver et al. (2021) (Table 1).

**TABLE 1:** Histopathologic score to assess wound healing.

​	score 0	score 1	score 2	score 3	score 4	score 5
Re-epithelization and epidermal thickness	Absent	Minimal	Partial	Moderate	Nearly complete	Complete and mature
Wound contraction	Excessive (considered detrimental)	Severe	Moderate	Mild	Minimal	Optimal (balanced contraction)
Granulation tissue maturation	Immature	Minimal maturation	Mild maturation	Moderate maturation	Advanced maturation	Fully matured
ECM remodelling	Absent	Minimal, disorganized	Mild, slightly organized	Moderate, partially organized	Abundant, mostly organized	Extensive, well-organized

### 2.7 Immunofluorescence investigations

Deparaffinization of the 5 µm thick sections were done using standard protocols. The sections were permeabilized with 0.2% Tween®20 (Sigma-Aldrich, France) solution in PBS for 30 min (10 min wash x 3). The sections were then blocked using 10% NGS (Normal Goat Serum, Invitrogen, UK)/5% BSA/0.3M Glycine (prepared in permeabilizing solution) for 1h. Antibodies against following markers were allowed to react overnight at 4 °C on the regenerated tissue, CD 31 (1:100), VEGF-A (1:500), CD 163 (1:100), IL-1 β (1:200), α- SMA (1:100) and albumin (1:200) on the scab (Table 2).

**TABLE 2:** List of antibodies.

Indicators	Primary antibodies (catalog no.)	Dilutions in 1% BSA solution
Angiogenesis	CD31 (ab119339, Abcam, UK)	1:100
	VEGF- A (ab1316, Abcam, UK)	1:500
Inflammation	IL-1β (Abcam, UK)	1:200
M2 macrophage	CD163 (ab156769, Abcam, UK)	1:100
Myofibroblasts and wound contraction	α- SMA (ab7817, Abcam, UK)	1:100
Wound exudate	Albumin (A6684, Abcam, UK)	1:200

After overnight incubation with the primary antibody, the slides were rinsed by PBS prior to addition of the secondary antibodies (Alexa 488-conjugated goat anti-mouse IgG (Cat no. A32723, Invitrogen, UK) and/or Alexa 594-conjugated goat anti-rabbit IgG (Cat no. A11012, Invitrogen, UK) at 1:800 dilution at room temperature for 1 h in the dark. The tissue sections were then counterstained for nuclei using the mounting medium with DAPI (ab104139, Abcam, UK). The images were taken under a fluorescence microscope (Olympus BX43, Japan) at ×20 magnification. Samples were incubated with only secondary antibodies as controls.

Images were captured by cellSens Imaging Software (Evident, Olympus Life Science Solutions, Japan) and semi-qualified by ImageJ. The background and exposure parameters were calibrated, and the cell counting was performed using ImageJ. Relative expressions between the groups were calculated for analysis.

### 2.8 Statistical analysis

Statistical analysis was done using SPSS statistical software (SPSS 31.0, SPSS, Inc. Chicago, IL, USA). All the tests were two tailed and p ≤ 0.05 was considered statistically significant. One-way repeated measures ANOVA (General Linear Models) with Bonferroni post-hoc tests were used across four within-subjects treatments (Empty, CG, BL and BL (GAS)) in individual experimental animals (n = 7).

## 3 Results

### 3.1 General assessment of the wound healing

The wound healing rates of different wound site conditions were evaluated in each animal. A gradual decrease in original wound size was shown over time in all studied groups (Figure 1). Clear contraction of wound was visible in the “empty” group at day 7, which was completely closed by day 14 (92.5% ± 2.11%). “Collagen (CG)” and “Bilayer (BL)” group showed signs of wound closure (85.8% ± 3.38% and 83.5% ± 1.92% respectively) with incomplete re-epithelization (histology) at day 14. The wound was significantly visible and was not completely closed at day 14 in “Bilayer -dual gene activated (BL (GAS))” group (wound closure at day 14 was 78.0% ± 2.08%). A thick scab with exudate was also prominently seen over “BL” and “BL (GAS)” group at day 14. Bonferroni-corrected pairwise comparisons showed “empty” differed significantly from “BL” (p < 0.05) and “BL (GAS)” (p < 0.01) both at day 7 and day 14 (Figure 1).

### 3.2 Histological evaluation characterizing the cell type and morphology of the regenerated tissue

**FIGURE 2:**
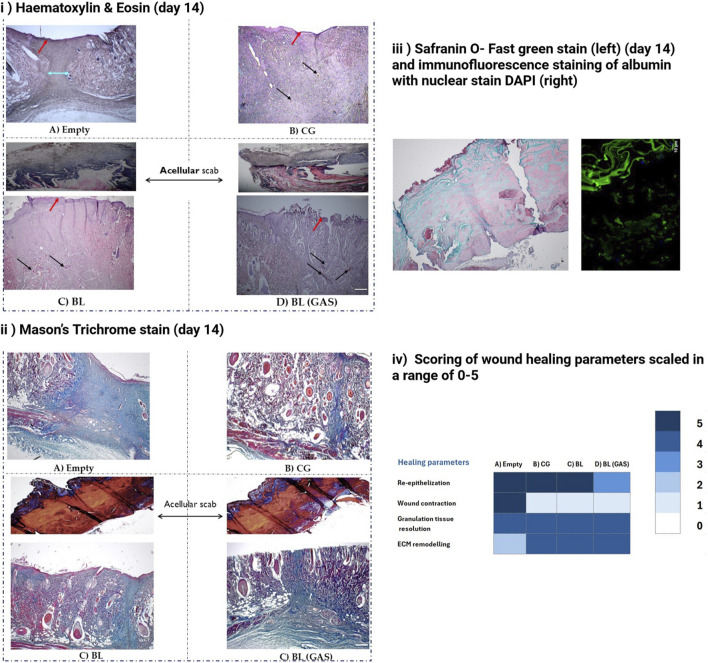
**(i)** H& E staining **(i)** At day 14, reepithelization (indicated by red arrows) was complete in “empty”, “CG” and “BL” groups. Sharp contraction of wound is visible in ‘empty’ group (indicated by cyan arrow). RBC infiltration was noted in scaffold groups (indicated by black arrows). Uniform layout of ECM remodelling protein is seen in “CG,” “BL,” “BL (GAS),” scale bar 50 μm. **(ii)** Mason’s trichrome staining shows that dense collagen remodelling (blue) in “empty” group. Dermal layer of the skin is prominent with cells (Red to pink stain of cytoplasm) in the “CG,” “BL” and “BL (GAS)” group. Incomplete reepithelization in “BL (GAS)” group is visible, scale bar 50 μm. **(iii)** Left- Safranin O- Fast green staining confirms that the scab is the outer chitosan-collagen layer of the bilayered scaffold. Chitosan takes the bluish to green stain and the collagen appears red to pink. No cells were detected in the scab; Right- Merged image of immunofluorescence staining of albumin with DAPI in the scabs from “BL” and “BL (GAS)” groups. **(iv)** Scoring of wound healing parameters based on histopathological evaluations using the modified criterion described by Arslantas et al. (2015), van de Vyver et al. (2021).

Histological evaluation with H&E and Masson’s trichrome stains demonstrated distinct differences between the “empty” and the scaffold groups (“CG,” “BL” and “BL (GAS)”) by day 14 of wound healing. In “empty” group, granulation tissue was largely resolved due to pronounced wound contraction (indicated by cyan arrow in Figure 2i), but extracellular matrix remodelling appeared non-uniform, likely as a direct consequence of this contraction process. In contrast, the scaffold groups (“CG,” “BL” and “BL (GAS)”) did not exhibit wound contraction; instead, progressive maturation of granulation tissue was seen. The uniform deposition of collagen was demonstrated by the even distribution of blue-stained fibers in the dermal layers in the scaffold groups on Masson’s trichrome-stained sections, while red to pink staining indicated the presence of cell cytoplasm Figure 2ii. By day 14, the epidermal layer had re-formed in the “empty,” “CG” and “BL” group, indicating re-epithelialization, although this process remained incomplete in the “BL (GAS)” group (depicted by red arrows in Figure 2i). Within scaffold groups (“CG,” “BL” and “BL (GAS)”), persistent infiltration of red blood cells was noted (depicted by black arrows in Figure 2i), along with marked immune cell infiltration in the “BL (GAS)” wounds.

The scab collected at day 14 from the “BL” and “BL (GAS)” wound sites were subjected to H&E, Masons trichrome and safranin O- fast green staining. Hairs were growing through the scab derived from the “BL” group. There were no traces of cells in the scabs. The scab obtained from both the BL (“BL” and “BL (GAS)”) groups showed positively stained for albumin (immunofluorescence), a major component in the tissue exudate (Figure 2iii). Summary of the wound healing parameters were scaled and are presented in Figure 2iv.

### 3.3 Immunofluorescence analysis

#### 3.3.1 Resolution of inflammation in the regenerated tissue differs between scaffold and empty group

**FIGURE 3:**
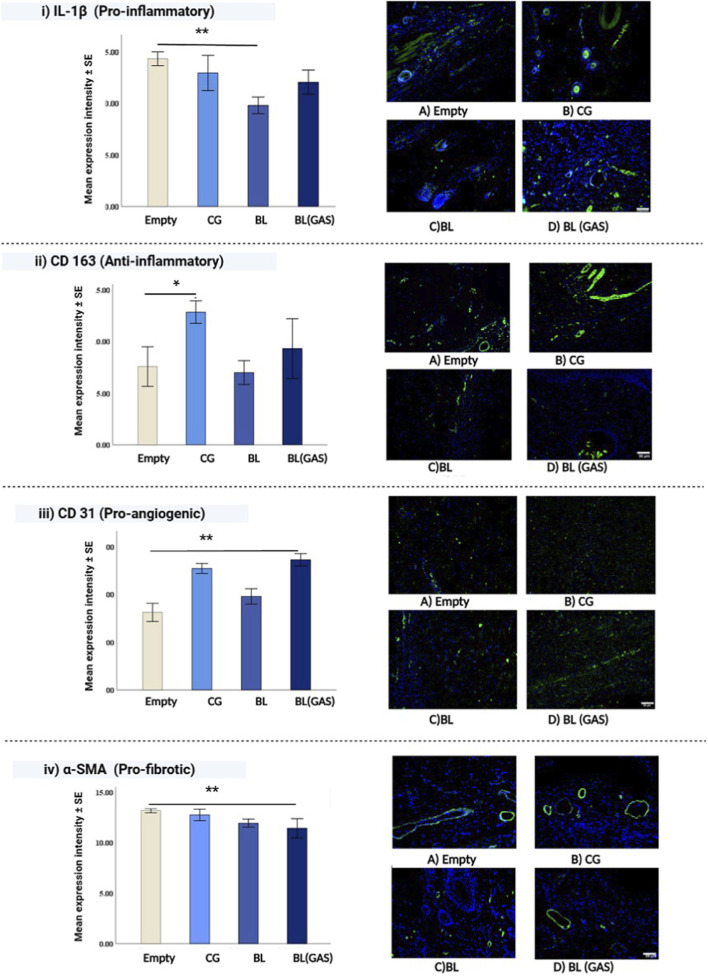
**(i)** Merged images of immunofluorescence staining of IL-1β (pro-inflammatory marker) with DAPI in all groups, IL-1β decreases in both the “BL” and “BL (GAS)” groups at day 14. IL-1 β (pro-inflammatory marker) significantly reduced in “BL” group when compared to “empty” group (One way ANOVA, p = 0.002); **(ii)** CD163 (M2 polarization, anti-inflammatory marker) expression was highest in “CG” group at day 14. The expression levels of CD163 are significantly higher in “CG” versus “empty” (p = 0.02) at day 14 of wound healing stage; **(iii)** CD31 (pro-angiogenic marker) was higher in “BL (GAS)” group as compared to “empty” (One way ANOVA, p < 0.01); **(iv)** α-SMA (pro-fibrotic marker) decreased significantly in “BL (GAS)” at day 14 as compared to “empty” group (One way ANOVA, p < 0.01); scale bar 50 μm, ** indicates p < 0.01 and * indicates p < 0.05.

IL-1β, a pro-inflammatory cytokine is noted to decrease in both bilayer scaffold (“BL” and “BL (GAS)”) groups, notably the decrease was more evident in the “BL” group (F = 10.811, p = 0.003). Post-hoc analysis indicated that the “BL” group had significantly lower expression of IL-1β at day 14 as compared to “empty” group (p = 0.002) (Figure 3i).

Resolving inflammatory phase is an important step in wound healing. M2 polarization is essential to transiently switch the pro-inflammatory environment to anti-inflammatory environment to proceed to proliferative phase of wound healing. CD163 (a surface marker for M2 macrophage) was expressed highest in “CG” group at day 14 and the *post hoc* analysis revealed that CD163 expression in “CG” group was significantly higher compared to “empty” (p = 0.02) (Figure 3ii).

#### 3.3.2 Regenerated tissue in the “scaffold” groups exhibited higher expression of CD31, a pro-angiogenic marker, with the highest levels observed in the pSDF-1α–activated scaffold group

Immunofluorescence analysis revealed that at day 14 angiogenesis was prominent in the “scaffolds” groups (“CG”, “BL,” and “BL (GAS)”) with the highest expression of CD31 in the “BL (GAS)”; however, its expression was not significantly different from the ‘CG’ and “BL” groups. Post- hoc analysis revealed that expression of CD-31 was higher in “BL (GAS)” as compared to “empty” group (p < 0.001) (Figure 3iii). Expression of VEGF did not differ significantly across the groups at day 14 (results not shown).

#### 3.3.3 Regenerated tissue in the scaffold groups showed reduced myofibroblast activity, with the pβKlotho-activated scaffold exhibiting the lowest α-SMA expression

Alpha smooth muscle actin (α-SMA) is expressed by the myofibroblasts in the granulation tissue, where its expression starts increasing around day 6 till day 12 post-injury, after which its levels decline. α-SMA decreases significantly in the “BL (GAS)” group as compared to the “empty” group at day 14 (p < 0.01), while there is no significant decrease in α-SMA when compared to other groups (Figure 3iv).

In summary, the “empty” group displayed complete re-epithelization of the wound which majorly closed by contraction. The other three scaffold groups (“CG”, “BL” and ‘BL (GAS)”) exhibited uniform wound closure. The bilayer scaffolds groups (“BL” and “BL (GAS)”) had lower levels of pro-inflammatory IL-1β as compared to “empty” and “CG” group. Angiogenesis was higher in all three-scaffold group (“CG,” “BL” and “BL (GAS)”) compared to “empty,” with the highest CD31 expression in the “BL (GAS)” group. Profibrotic marker α-SMA was lowest in bilayer scaffolds groups (“BL” and “BL (GAS)”) with the least in the “BL (GAS)” group.

## 4 Discussion

The management of chronic wounds necessitates a comprehensive and holistic strategy in which the three-dimensional tissue defect must be addressed using advanced biomaterials that are biocompatible, structurally stable, biodegradable, and capable of sustaining a moist microenvironment while simultaneously absorbing excess exudate. Such biomaterials not only provide a supportive matrix for cellular adhesion, proliferation, and differentiation, thereby guiding tissue regeneration, but also serve as a protective scaffold for orchestrating reparative processes. Despite these advances, effective control of microbial colonization and infection within the wound bed remains a persistent and critical challenge, often impeding successful healing outcomes (Freedman et al., 2023). Chitosan based dressings, formulations and 3D scaffolds have extensively been researched for bone, cartilage, neural and skin regeneration of which a few has entered into clinical trials primarily for its antimicrobial and anti-inflammatory role (Pramanik et al., 2024; Feng et al., 2021; Rajinikanth et al., 2024).

Taking this into consideration, the tissue engineering team at TERG Dublin developed a bilayer chitosan- collagen (dermal collagen–glycosaminoglycan (CG) porous layer combined with an epidermal chitosan-collagen film layer) which displayed wound healing and antimicrobial properties *in vitro* (McGrath et al., 2023). As a continuation to the previous *in vitro* findings, in this study we tested the *in vivo* wound healing ability of this novel bilayered scaffold and assessed if targeted gene activated counterparts of this scaffold would augment its wound healing potential.

**FIGURE 4:**
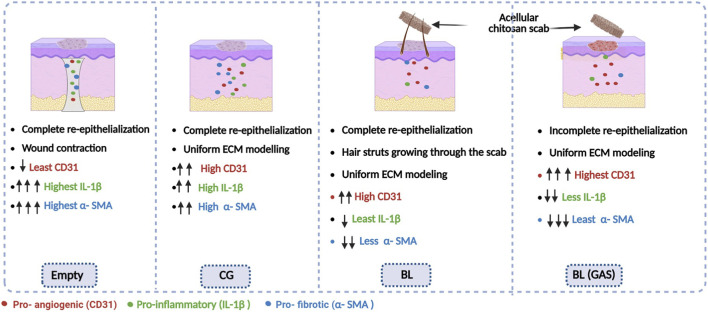
Schematic overview of wound healing attributes in “empty,” “CG,” “BL,” and “BL” (GAS) groups. The “empty” wound closed by sharp contraction; compared to other groups, “empty” exhibited least pro-angiogenesis (CD 31) and highest pro-inflammatory (IL-1β) and pro-fibrotic (α-SMA) expression; “CG” group showed complete re-epithelialization and compared to “empty,” the day 14 wound had more pro-angiogenesis (CD 31), similar high pro-inflammatory (IL-1β) and a bit lower pro-fibrotic (α-SMA) expression; “BL” group had a completely re-epithelialized wound with a protective acellular chitosan scab which detached easily with hair growing through the scab. Compared to “empty” and “CG” group, the regenerated wound showed fair indication of pro-angiogenesis (CD 31), least pro-inflammatory (IL-1β) and lower pro-fibrotic (α-SMA) marker; “BL (GAS)” regenerated wound did show highest angiogenesis marker (CD 31), lower pro-inflammatory (IL-1β) (green dots) and least pro-fibrotic (α-SMA) marker, however the wound did not show complete reepithelialization.

A comprehensive schematic overview of the wound repair in “empty,” “CG,” “BL” and “BL (GAS)” is presented in Figure 4.

### 4.1 The bilayer chitosan-collagen scaffold exhibited a wound healing trajectory comparable to established acellular dermal matrices (ADMs)

We noted that in both bilayer chitosan- collagen groups (“BL” and “BL (GAS)”), a thick scab detached effortlessly at day 14. However, in the “bilayer gene activated (BL (GAS))” group, the scab revealed the underlying wound that was not fully re-epithelialized, whereas in “bilayer (BL)” group, the scab exposed completely healed and re-epithelialized tissue. Interestingly we noted hair growing through the scab in “BL” group, which can be indicative of completely repaired and regenerated wound (Chuong, 2007); however, this finding needs further investigation (Figure 4). Histochemical staining indicated that these scabs were the upper chitosan- collagen film layer of the bilayered scaffolds, prominently filled with proteinaceous (albumin-rich) exudate (as seen in immunofluorescence staining). While chitosan has been referred to as biodegradable in many applications such as nanoparticle drug delivery, hydrogels for cutaneous wounds, our study indicates that the degradation rate of chitosan is much slower in a cutaneous wound (Saputra, 2025). Chitosan is reportedly having a lower biodegradation rate, and attempts have been made to functionalize the chitosan (mainly with imine linkage) to slow its biodegradation rate so that they can be eliminated from the wound naturally and thereby reducing dressing related trauma (Lungu et al., 2021; Movaffagh et al., 2022; Hasanin et al., 2022). Our data reinforce these findings by demonstrating that, although chitosan film does not undergo complete integration at the cutaneous wound site, its persistence may nonetheless contribute positively to sustained healing by providing a protective layer. The formation of thick wound scab can be attributed to the slow degradation rate of chitosan, and this property can be harnessed clinically for wound healing treatments without the necessity of a traumatic dressing removal; thereby enhancing the therapeutic potential of using the bilayered scaffolds as a dressing material to treat complex wounds such as burn and chronic wounds such as diabetic foot ulcers (DFU). This delamination parallels the non-integrative outer layers of commercial acellular dermal matrices (ADMs), supporting effective dermal regeneration without permanent incorporation. For instance, Integra® Dermal Regeneration Template comprises a bilayer structure featuring an outer silicone layer that remains non-integrative, serving as a protective barrier against infection and moisture loss (Bassetto et al., 2025). Similarly, NovoSorb® Biodegradable Temporizing Matrix (BTM) consists of an inner biodegradable polyurethane matrix paired with an outer non-biodegradable polyurethane sealing membrane. Post-integration, the sealing membrane is removed, yielding a vascularized neodermis primed for secondary interventions such as grafting (He et al., 2025). In alignment with these mechanisms, our bilayer chitosan-collagen scaffold demonstrated delamination of the upper chitosan layer.

### 4.2 Biomaterials that elicit no inflammatory signals while actively suppressing pro-inflammatory responses hold substantial promise for chronic wound management

The bilayer chitosan- collagen groups (“BL” and “BL (GAS)”) exhibited reduction in IL-1β expression levels as compared to “empty” and the “CG” group, highlighting the anti-inflammatory potential of the upper chitosan film and suggesting its advantageous role in modulating the wound healing environment. The orderly and timely advancements through the four phases of wound healing—haemostasis, inflammation, proliferation, and remodelling—is critical for effective tissue repair. In chronic wounds, this process is disrupted by prolonged inflammatory phase and insufficient cellular proliferation (Darwin and Tomic-Canic, 2018); while in the context of biomaterial implantation, the foreign body response can further exacerbate inflammatory activity. Hence it is imperative that the engineered biomaterials not only recapitulate native tissue properties, but it should also actively modulate the immune response to support regeneration (Hortensius and Harley, 2016; Xu et al., 2021). The observed reduction in IL-1β expression levels within the “bilayer chitosan- collagen” groups as compared to “empty” and the “CG” groups alone highlights the anti-inflammatory potential of chitosan, suggesting its advantageous role in modulating the wound healing environment. This property could be strategically leveraged in relevant wound models to further enhance tissue repair by mitigating excessive inflammation. Beyond IL-1β, chitosan’s capacity to downregulate other pro-inflammatory cytokines such as TNF-α, combined with its intrinsic antimicrobial properties, establishes it as a highly promising material for the treatment of complex wounds, including diabetic foot ulcers (DFUs); however the exact mechanism of its anti-inflammatory action is not yet explored (Zosangpuii, 2024). Another study which assessed the wound healing ability of chitosan membrane on full- thickness skin splits on 54 rats showed high serum levels of IL-4 day 7 in the treatment groups indicating a shorter inflammatory phase (Nordback et al., 2015). Curcumin-loaded chitosan nanoparticles showed similar results in promoting wound healing in diabetic rat model by mitigating the macrophage cells-mediated inflammation (Li et al., 2019). These findings collectively suggest that chitosan-based biomaterials can effectively attenuate excessive inflammation and promote tissue regeneration in diverse wound models.

### 4.3 Neodermis quality: balancing vascularization and fibrosis

CD31 is a well-known marker of angiogenesis and is primarily expressed on endothelial cells. Our findings demonstrated that all three scaffold groups (“CG,” “BL” and “BL (GAS)”) exhibited higher CD31 expression relative to the “empty” control, underscoring their intrinsic ability to support cell attachment, migration and angiogenic processes within the wound microenvironment. Importantly, functionalization of the bilayer chitosan- collagen with the chemoattractant pSDF-1α in the “BL (GAS)” group, further augmented this effect, consistent with its reported role in mobilizing endothelial progenitor cells and enhancing vascular ingrowth (Raftery et al., 2024).

Towards the mid phase of the wound healing, fibroblasts differentiate into contractile myofibroblasts under the influence of growth factors like TGF-β, α-SMA expression enhances contributing to wound contraction and granulation tissue formation. As the wound heals (day 14), these myofibroblasts undergo apoptosis and this timely regulation where α-SMA diminishes helps in avoiding excessive fibrosis and abrupt wound contraction (Singer, 2022; Younesi et al., 2024). We noted in our study that α-SMA were higher in the “empty” group which underwent predominantly rapid contraction-mediated closure as compared to other scaffold groups. It was interesting to note that the α-SMA levels were lower in both the bilayer scaffold groups (“BL” and “BL (GAS)”). The expression of α-SMA was significantly lower expression in the “BL (GAS)” group as compared to the “empty” group, which was functionalized with pSDF1α and pβKlotho. β-Klotho mediates anti-fibrotic effects in skin primarily by modulating FGF signalling to promote basement membrane integrity, enhancing elastin matrix deposition and reducing fibrotic markers such as α-smooth muscle actin (α-SMA) (Laiva et al., 2024; Suku et al., 2020; Lin et al., 2025). We noted general decrease in α-SMA in all scaffold groups with significant decrease in ‘BL (GAS)’ group, delineating the anti-fibrotic role of pβKlotho activated bilayer chitosan- collagen.

As demonstrated in previous reports by our team, in this study all the three scaffold groups (“CG,” “BL” and “BL (GAS)”) displayed wound closure and uniform layout of the extracellular matrix (ECM) protein with complete resolution of the granulation tissue at the end of day 14 (Das et al., 2025).

### 4.4 Gene-activated bilayer scaffold provided targeted therapeutic delivery; however, it demonstrated delayed wound healing kinetics

Although the dual gene activated scaffold ‘BL (GAS)’ functionalized with pSDF1α and pβKlotho demonstrated some advantage over other groups (“empty,” “CG” and “BL”) with enhanced angiogenesis and reduced pro-fibrotic markers, the neodermis failed to achieve complete re-epithelization at day 14. This necessitates further investigation to determine whether pSDF1α and pβKlotho exhibit distinct functional roles, or if the antifibrotic pβKlotho should be activated specifically during the mid-phase of wound healing (Farokhi et al., 2016).

It was interesting to note that the bilayer ‘BL’ group by itself without any gene activation, exhibited superior wound healing ability compared to other groups, characterized by facile delamination of the upper chitosan layer and robust integration of the dermal collagen layer with maturation of granulation tissue, complete re-epithelialization, reduced pro-inflammatory milieu, reduced pro-fibrotic and fair degree of angiogenesis at the day 14 wound healing assessment.

The next steps would be to bridge the gap between experimental results and real-world clinical challenges in wound management by examining in a diabetic and/or infectious wound model to mimic post-debridement infected non-union ulcers to study scaffold performance in bacterially challenged environments, which being the most challenging situation in treating chronic wounds. Future studies should investigate chitosan degradation kinetics within the wound environment to better understand bioactive release and matrix remodelling. The single time-point assessment (day 7) limits evaluation of healing rate dynamics and late-stage tissue remodelling across multiple time points (days 3, 7, 14, 28). Lack of randomized wound assignment within animals represents an additional methodological limitation.

## 5 Conclusion

In conclusion, these chitosan-based bilayered scaffolds represent an advanced next-generation tissue engineering biomaterial wherein the upper chitosan-collagen layer exhibits minimal adhesion to the wound surface, allowing for natural and atraumatic detachment during the healing process. Concurrently, the underlying collagen-chitosan layer facilitates tissue regeneration within the wound bed by promoting cell attachment and proliferation while mitigating inflammation, as evidenced by a reduction in IL-1β expression. This bilayered structure thus creates an optimal microenvironment for enhanced wound healing through combined anti-inflammatory and regenerative mechanisms. Incorporation of gene activation strategies to promote pro-angiogenic and anti-fibrotic responses further augments its therapeutic efficacy, highlighting the potential of chitosan- collagen programmed scaffolds as a promising approach for the treatment of chronic, non-healing wounds.

## Data Availability

The raw data supporting the conclusions of this article will be made available by the authors, without undue reservation.
